# Miscellany

**Published:** 1899-12

**Authors:** 


### MISCELLANY.

The Physicians' Pocket Manual, is the name of a compact little book sent out to the medical profession by Messrs. Parke Davis & Co., of Detroit. It contains a fund of information about pharmaceutical and biological remedies, fifty-eight pages of which are devoted to a property and dose list. The manual further includes tables of metric equivalents, a table for making solutions, a complete index, an exhaustive list of botanical synonyms, besides many items of valuable information in the form of "notes."

In addition it is not out of place to note that it presents an array of over thirty distinct lines of remedial agents, comprising nearly 5,000 separate products. All this bears testimony to the growth of this great house, the magnitude of the scientific work it carries on, and the great volume of business it transacts. Its methods have won the confidence of the medical profession and it is easy to discover that it is the purpose of all who are conducting its affairs to strive to maintain this esteem.

